# Anwuligan promotes the transition of the hair follicle cycle via the Wnt/β-catenin signaling pathway

**DOI:** 10.3389/fphar.2025.1704083

**Published:** 2026-01-21

**Authors:** Fang Li, Leyao Zhang, Yan Li, Xiang Zheng

**Affiliations:** 1 The Center Laboratory, Changzhi Medical College, Changzhi, Shanxi, China; 2 Basic Medicine, Changzhi Medical College, Changzhi, Shanxi, China

**Keywords:** anwuligan, cycle transition, hair follicle, hair regeneration, traditional Chinese medicine, Wnt/β-catenin

## Abstract

**Background:**

Hair loss is a significant dermatological condition and hair regeneration depends on orderly cycle of hair follicle activity. Traditional Chinese Medicine (TCM) suggests that BaoHeWan can improve dry and sparse hair, but the molecular mechanisms behind this effect are not well known.

**Methods:**

This study utilizes network pharmacology, phenotype observation, H&E staining, transcriptomics and molecular biology methods to investigate how anwuligan promote hair regeneration. demonstrated that anwuligan promoted hair regeneration in model mice.

**Results:**

Using network pharmacology analysis and molecular docking, anwuligan was found to be a major active ingredient in BaoHeWan, potentially acting by binding to CTNNB1. Transcriptomics analysis of mice treated with anwuligan, complemented by cellular validation, demonstrated that anwuligan induces upregulation of Ctnnb1 expression, activates the Wnt/β-catenin signaling pathway, promotes the proliferation of hair follicle stem cells (HFSCs), and facilitates the progression of the hair follicle cycle.

**Conclusion:**

These results elucidate the molecular mechanisms underlying anwuligan's facilitation of hair regeneration and offer a scientific foundation for the utilization of TCM in the treatment of hair loss.

## Introduction

Hair loss is one of the most prevalent skin diseases worldwide, affecting a wide range of people and showing a trend toward younger individuals. The essence of hair loss is the disruption of the hair follicle cycle or regeneration disorders, and restoring the regenerative ability of hair follicles is the core strategy for treating hair loss (Alonso and Fuchs, 2006; Schneider et al., 2009). The hair follicle cycle includes three phases: anagen, catagen, and telogen (Müller-Röver et al., 2001). Hair regeneration depends on the normal and orderly cycle of hair follicle activity (Wang et al., 2023).

Traditional Chinese medicine (TCM), with a history spanning thousands of years, plays a vital role in the prevention and treatment of various major chronic diseases (Qian et al., 2023). However, its pharmacological basis and target mechanisms require further clarification. The occurrence of phenomics research methods such as transcriptomics and metabolomics presents a favorable opportunity for the advancement of TCM (Zhang et al., 2024). These research methods can deeply enhance the level of human physiological and pathological cognition, explain the scientific connotation of Chinese medicine syndromes, and reveal the effective material basis and functional mechanisms of herbal medicines, thereby promoting its clinical translation (Zhang et al., 2024). TCM theory to dialectical treatment believes that BaoHeWan can improve dry and sparse hair by promoting hair regeneration, but their potential molecular mechanisms still lack in-depth research.

*Forsythia suspensa* (forsythia), a key botanical in the traditional Chinese formula BaoHeWan, is a widely distributed deciduous shrub and a fundamental component of Chinese medicine. In recent years, it has garnered significant research interest due to its broad pharmacological activities, including antioxidant, anti-inflammatory, antibacterial, antiviral, and immuneregulatory effects (Zeng et al., 2020; Li et al., 2025). Among its bioactive constituents, the monomeric lignan anwuligan has been identified to possess notable anti-apoptotic, antioxidant, and anti-inflammatory properties (Zhang et al., 2019; Li et al., 2020). These activities underpin its demonstrated efficacy in mitigating conditions such as intestinal ischemia–reperfusion injury (Lin et al., 2021). In addition, anwuligan has been recognized as a novel Janus kinase 1 (JAK1) inhibitor, with potential in suppressing the growth of non-small-cell lung cancer (Xie et al., 2021). However, its relationship with hair loss remains virtually unknown.

The inter- and intracellular signaling pathways, including, but not limited to, sonic hedgehog (shh), Wnt/β-catenin, bone morphogenetic protein (BMP), transforming growth factor (TGF)-β, and notch signaling, have been implicated in the hair regeneration process (Choi, 2020). The Wnt/β-catenin (encoded by CTNNB1) signaling pathway is one of the core pathways that regulate hair follicle development, hair follicle cycle, and hair follicle regeneration (Schmidt-Ullrich and Paus, 2005; Fuchs, 2007; Zhang et al., 2009). Its activation or inhibition directly affects the activation of hair follicle stem cells (HFSCs) and hair growth (Choi et al., 2013). The diabetic condition reduced both hair regrowth and regeneration with suppression of the Wnt/β-catenin signaling pathway (Ryu et al., 2022).

In this study, we combined TCM theory to dialectical treatment with network pharmacology analysis to examine the active components in BaoHeWan. We found that component anwuligan in forsythia may activate the Wnt/β-catenin signaling pathway by binding to CTNNB1. The Wnt/β-catenin signaling pathway is the core pathway that regulates hair follicle development, hair follicle cycle, and hair follicle regeneration. Based on this, we further investigated the effect of anwuligan on hair follicle regeneration and its molecular mechanisms. This study will enrich the potential molecular mechanisms of TCM theory to dialectical treatment, providing a reference for the development of targeted drugs for the treatment of hair loss.

## Materials and methods

### Animals

Animal work in this manuscript was approved by the Experimental Animal Manage Committee of Changzhi Medical College (DW2024025). For this study, 6–7-week female C57BL/6 strain mice were purchased from Home-SPF (Beijing) Biotechnology Co., Ltd. Following a 1-week acclimatization period in the laboratory, the mice were subjected to a hair removal procedure. Subsequently, the mice received gavage administration of anwuligan at dosages of 0 mg/kg, 1 mg/kg, 2 mg/kg, and 4 mg/kg over a duration of two consecutive weeks, until the onset of hair regrowth was observed. Each group comprised four biological replicates. Mice were housed in a temperature-controlled room with a 12-h light/dark cycle, 40%–60% humidity, and *ad libitum* access to water and food.

### Sample collection

Upon the development of a hair coat on the dorsal region of the mice, skin tissue samples were obtained from this area. A portion of the tissue was preserved in an RNA stabilization solution for transcriptome sequencing and RNA extraction, while the remaining portion was fixed in 4% paraformaldehyde to facilitate the preparation of paraffin-embedded sections.

### H&E staining

Formalin-fixed and paraffin-embedded mouse skin specimens were cut into 4-μm sections, stained with hematoxylin and eosin (H&E), and observed using light microscopy.

### RNA extraction and skin tissue transcriptome analysis

Total RNA was extracted from the three individual dorsal skin tissues of the control group (0 mg/kg) and medium-concentration addition group (2 mg/kg). After quality checking, ∼1 µg total RNA for each sample was used to construct the RNA-seq transcriptome library according to the protocol (Illumina® Stranded mRNA Prep). A total of six libraries (control group and medium-concentration addition group, each with three replicates) were constructed and performed on the NovaSeq 6000 platform using the NovaSeq reagent kit. The RNA purification, reverse transcription, library construction, and sequencing were performed at Shanghai Biotechnology Corporation Co., Ltd. (Shanghai, China).

According to the transcripts per million reads (FPKM) method, the gene expression levels of transcript were calculated to further identify the differential expression genes (DEGs) between two groups. DEGs were screened using DESeq2 with |log_2_FC|≥1 and *P* < 0.05 (Love et al., 2014).

### GO and KEGG enrichment analyses

DAVID 6.8 (https://david.ncifcrf.gov/) and KOBAS 3.0 (http://kobas.cbi.pku.edu.cn/kobas) were used for Kyoto Encyclopedia of Genes and Genomes (KEGG) and Gene Ontology (GO) enrichment analyses. GO and KEGG enrichment analyses of DEGs were performed to investigate the underlying functions and some vital pathways.

### RNA isolation and qRT-PCR

Total RNA from the tissues and cells was extracted using the TRIzol method (Takara, Japan). The concentration and purity of the RNA samples were examined using NanoDrop 2000 (Thermo Fisher Scientific, United States). Reverse transcription was performed using the All-in-one First-Strand cDNA synthesis kit Ⅱ (Sevenbio, Beijing) for cDNA amplification. Quantitative real-time PCR (qRT-PCR) was performed using 2× sybr Green qPCR MasterMix Ⅱ (Sevenbio, Beijing) and the ABI StepOnePlus™ real-time PCR system (Applied Biosystems, United States), with β-actin as the internal reference. The primers used for these experiments are shown in Table1. The comparative threshold cycle (Ct) method was used to measure the relative expression, where 2^−ΔΔCt^ represents the fold change in expression, as previously described (Livak and Schmittgen, 2001).

**TABLE 1:** Primer sequences used in qRT-PCR.

Gene name	Forward (5′–3′)	Reverse (5′–3′)
*Krt18*	CAG​CCA​GCG​TCT​ATG​CAG​G	CCT​TCT​CGG​TCT​GGA​TTC​CAC
*Dsg4*	TCT​CCT​AGT​ACA​GCC​TGC​TT	AGT​GGT​CTC​TCC​AAG​TCT​TC
*Fgf5*	CCC​ACG​AAG​CCA​GTG​TGT​TA	ATC​GCG​GAC​GCA​TAG​GTA​TT
*Dkk4*	CGA​AGG​GAA​ATT​CTG​CTT​AGC​G	TCA​TTC​ACA​CAG​ACC​GTT​CCT
*Smad6*	GTT​GCA​ACC​CCT​ACC​ACT​TC	GGA​GGA​GAC​AGC​CGA​GAA​TA
*Rspo4*	CTC​GCC​CTG​TAC​CGA​AGG​A	CAC​TTG​CCG​TAC​TGA​CGG​AT
*Hey2*	AAG​CGC​CCT​TGT​GAG​GAA​AC	GGT​AGT​TGT​CGG​TGA​ATT​GGA​C
*Sox2*	ATG​AAC​GGC​TGG​AGC​AAC​GGC​A	TCA​CAT​GTG​CGA​CAG​GGG​CAG​T
*Sox11*	CGA​GCC​TGT​ACG​ACG​AAG​TG	AAG​CTC​AGG​TCG​AAC​ATG​AGG
*Lgr6*	GTA​TGA​ACA​ACC​TCA​CGG​AGC	TTG​GAG​GCC​AGA​GAA​TGC​C
*Tbx15*	TAG​CAG​TGA​TCT​TTC​ACC​CAC​C	GCA​AAG​GGG​TTT​CGG​TCA​ATT​T
*Twist2*	CGT​CTC​AGC​TAC​GCC​TTC​TC	CCA​GGT​GCC​GAA​AGT​CAC​AG
*Ctnnb1*	ATG​GAG​CCG​GAC​AGA​AAA​GC	CTT​GCC​ACT​CAG​GGA​AGG​A
*β-Actin*	GGC​TGT​ATT​CCC​CTC​CAT​CG	CCA​GTT​GGT​AAC​AAT​GCC​ATG​T

### Cell culture and transfection

HFSCs and the culture supernatant of HFSCs were purchased from Wuhan Pricella Biotechnology Co., Ltd., China. HFSCs were seeded in 6-well plates and maintained in complete medium. Once HFSCs reached 80%–90% confluency, the cells were treated with different concentrations of anwuligan (0 μmol/L and 10 μmol/L). Following a 48-h incubation period post-transfection, the HFSCs were utilized for qRT-PCR, CCK-8, MTT, and EdU assays.

### CCK-8 assay

The obtained anwuligan-treated HFSCs were inoculated into 96-well plates at a density of 2.0 × 10^3^ cells per well. After 0 h, 24 h, 48 h, 72 h, and 96 h of inoculation, the CCK-8 reaction solution was added according to the instructions of the CCK-8 kit (Beyotime, Shanghai, China), and the absorbance value at the wavelength of 450 nm was detected using an TECAN reader after incubation at 37 °C for 1 h.

### EdU assay

The obtained anwuligan-treated HFSCs were inoculated into 24-well plates at a density of 2.5 × 10^5^ cells per well. An equal volume of 2× EdU working solution was added to the culture medium and incubated for 2 h. After removing the culture medium following EdU labeling of the cells, they were fixed using 1 mL of 4% paraformaldehyde at room temperature for 15 min. After washing and permeabilizing, 100 µL click reaction solution was added to each well, followed by incubation at room temperature in the dark for 30 min; then, the cell nuclei were stained with Hoechst 33342. Finally, the samples were observed under a fluorescence inverted microscope.

### MTT assay

The obtained anwuligan-treated HFSCs were inoculated into 96-well plates at a density of 2.0 × 10^3^ cells per well. An aliquot of 10 μL of 5 mg/mL MTT solution was added to each well and incubated in the cell culture incubator for 4 h. Then, 100 μL of formazan-dissolving solution was added, gently mixed, and incubated in the cell culture incubator for 3–4 h. An ELISA reader was used to measure the absorbance value at the wavelength of 570 nm.

### Western blot analysis

Total protein extraction and Western blotting were performed in accordance with the manufacturers’ instructions for the reagents. The primary antibodies used were anti-β-catenin (dilution ratio 1:5,000, Proteintech, Wuhan, China, Cat. No.: 51067-2-AP) and anti-β-actin (dilution ratio 1:2000, ZenBio, Chengdu, China, Cat. No.: T200068-8F10). Goat anti-rabbit IgG–HRP (dilution ratio 1:5,000, Biosharp, Shanghai, China, Cat. No.: BL003A) was used as a secondary antibody.

### Immunofluorescence

The immunofluorescence protocol was carried out as described previously (Ge et al., 2025). Anti-β-catenin (dilution ratio 1:200, Proteintech, Wuhan, China, Cat. No.: 51067-2-AP) as primary antibody and goat anti-rabbit IgG–HRP (dilution ratio 1:200, Servicebio, Wuhan, China, Cat. No.: GB25303) as secondary antibody were used. Hoechst 33342 was used for nuclear counterstaining.

### Statistical analysis

All experimental data were expressed as mean ± standard deviation (x ± s), and comparisons between two groups were made using one-way ANOVA. *P* < 0.05 was considered statistically significant. The results were statistically analyzed and visualized using GraphPad Prism 9.0 software.

## Results

### Network pharmacology analysis of BaoHeWan

**FIGURE 1:**
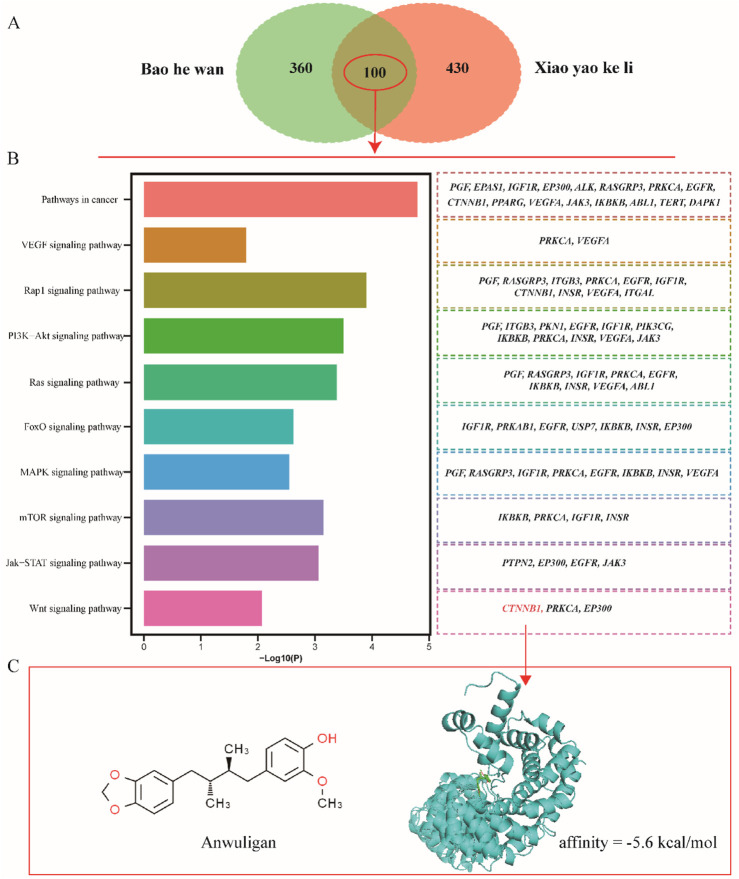
Results of network pharmacology and molecular docking. **(A)** Target genes of BaoHeWan and Xiaoyaokeli. **(B)** KEGG enrichment analysis of overlapping genes. **(C)** Molecular docking result of anwuligan and CTNNB1.

In ETCM 2.0, a total of 530 target genes for BaoHeWan were screened. To increase the credibility of the target genes, we conducted target gene analysis on Xiaoyaokeli, another TCM that can treat dry and sparse hair, and we screened a total of 460 target genes. Of these, 100 genes overlapped between treatments (Figure 1A). To verify the functions of these overlapping genes, we conducted KEGG enrichment analysis and found that these genes were significantly enriched in the VEGF signaling pathway, FoxO signaling pathway, MAPK signaling pathway, mTOR signaling pathway, and Wnt signaling pathway (Figure 1B). Our findings indicate that the essential gene *Ctnnb1*, which plays a pivotal role in the development and regeneration of hair follicles, exhibits a significant enrichment within the Wnt signaling pathway. CTNNB1 mainly exerts its function by binding to anwuligan. Additionally, we performed molecular docking studies involving CTNNB1 and anwuligan, which revealed a moderate binding affinity between the two entities (affinity = −5.6 kcal/mol) (Figure 1C). The above results indicate that anwuligan may promote the regeneration of hair follicles by activating the Wnt/β-catenin pathway via binding to CTNNB1.

### Anwuligan promotes the regeneration of hair follicles

**FIGURE 2:**
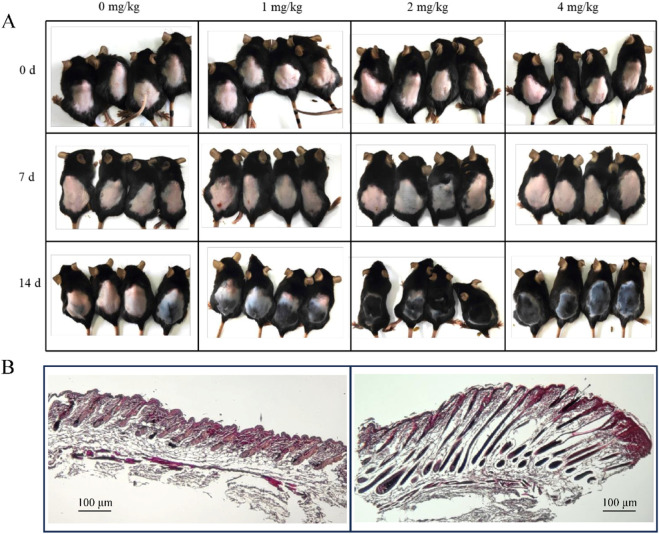
Anwuligan can promote mouse hair growth and the growth of mouse hair follicles. **(A)** Photographs of hair removal areas were taken on days 0, 7, and 14 after treatment with 0 mg/kg, 1 mg/kg, 2 mg/kg, and 4 mg/kg of anwuligan. **(B)** H&E staining of anwuligan 0 mg/kg and 2 mg/kg groups (left, 0 mg/kg; right, 2 mg/kg; H&E staining, ×100, scale bar = 100 µm).

Based on the similarities between animal and human skin, various animal models have been used in hair regeneration research. To functionally validate the above mechanistic insight, we assessed the hair regeneration efficacy of anwuligan in a mouse model. In this study, C57BL/6 mice treated for back hair removal were administered anwuligan via gavage at doses of 0 mg/kg, 1 mg/kg, 2 mg/kg, and 4 mg/kg, and hair growth on the backs of C57BL/6 mice was observed 14 days after gavage. The hair growth status is shown in Figure 2A. Overall, the addition of anwuligan accelerated the growth of dorsal hair in C57BL/6 mice, with the most noticeable effect in the 2 mg/kg group. The morphological structure of hair follicles in the anwuligan 0 mg/kg and 2 mg/kg groups was further investigated using H&E staining. The staining results indicated that hair follicles in the anwuligan 2 mg/kg group developed faster and exhibited a complete follicular structure (Figure 2B). This result confirms the promotional effect of anwuligan on hair regeneration in C57BL/6 mice.

### Effects of anwuligan on skin tissue transcriptome

**FIGURE 3:**
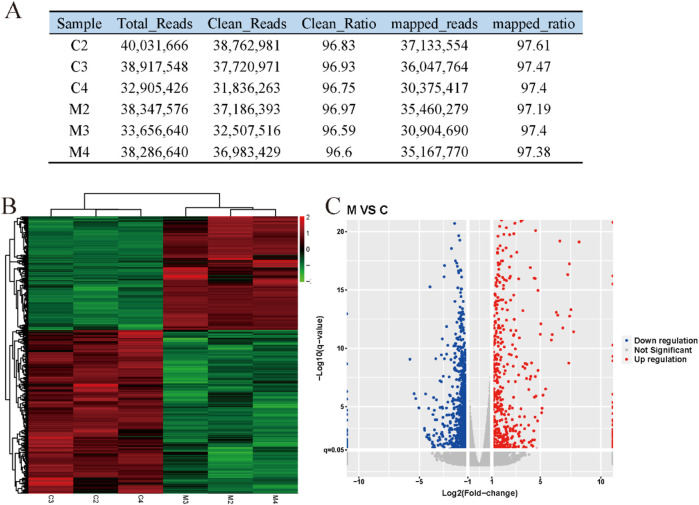
Differentially expressed genes between the medium-concentration anwuligan treatment group and the control group. **(A)** RNA-seq data quality control and mapping. **(B)** Heatmap of differentially expressed genes. **(C)** Volcano plot of differentially expressed genes. Note: M, 2 mg/kg anwuligan treatment; C, 0 mg/kg anwuligan treatment. M1, M2, and M3 represent the three biological replicates of 2 mg/kg anwuligan treatment; C1, C2, and C3 represent the three biological replicates of 0 mg/kg anwuligan treatment.

To further investigate the molecular mechanism of anwuligan in promoting hair regeneration in mice, the dorsal skin of C57BL/6 mice in the 0 mg/kg and 2 mg/kg addition groups of anwuligan was collected for transcriptome sequencing in this study. The results of RNA-seq data quality control and mapping are shown in Figure 3A. First, clustering analysis indicated high similarity among three replicates of the 0 mg/kg and 2 mg/kg addition groups of anwuligan (Figure 3B). A total of 1,638 differential genes were obtained through differential analysis, including 672 upregulated genes and 966 downregulated genes (Figure 3C).

**FIGURE 4:**
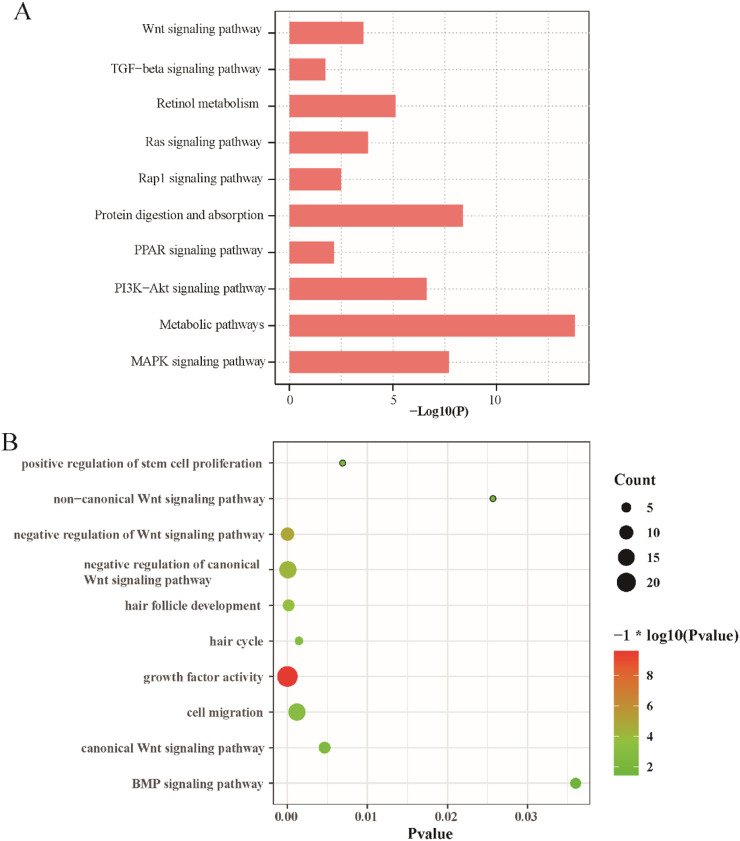
KEGG and GO enrichment analyses of DEGs between the medium-concentration anwuligan treatment group and the control group. **(A)** KEGG annotation. **(B)** GO annotation. Note: M, 2 mg/kg anwuligan treatment; C, 0 mg/kg anwuligan treatment.

To better understand the biological processes and signaling pathways modulated by anwuligan, GO and KEGG functional enrichment analyses were performed on the 1,638 DEGs. The DEGs were significantly enriched in metabolic pathways, protein digestion and absorption, retinol metabolism, MAPK signaling pathway, PI3K–Akt signaling pathway, Wnt signaling pathway, PPAR signaling pathway, and TGF-beta signaling pathway (Figure 4A). Concurrently, GO analysis of biological processes showed significant enrichment of the canonical Wnt signaling pathway, non-canonical Wnt signaling pathway, BMP signaling pathway, hair follicle development, hair cycle, cell migration, and positive regulation of stem cell proliferation (Figure 4B). Despite the enrichment of several hair growth-related pathways, we focused on validating the Wnt/β-catenin pathway due to its established role in hair follicle biology, the specific enrichment of canonical Wnt signaling, and the strong representation of its target genes.

### Validation of RNA-seq results and the expression analysis of Wnt/β-catenin signaling pathway-related genes

**FIGURE 5:**
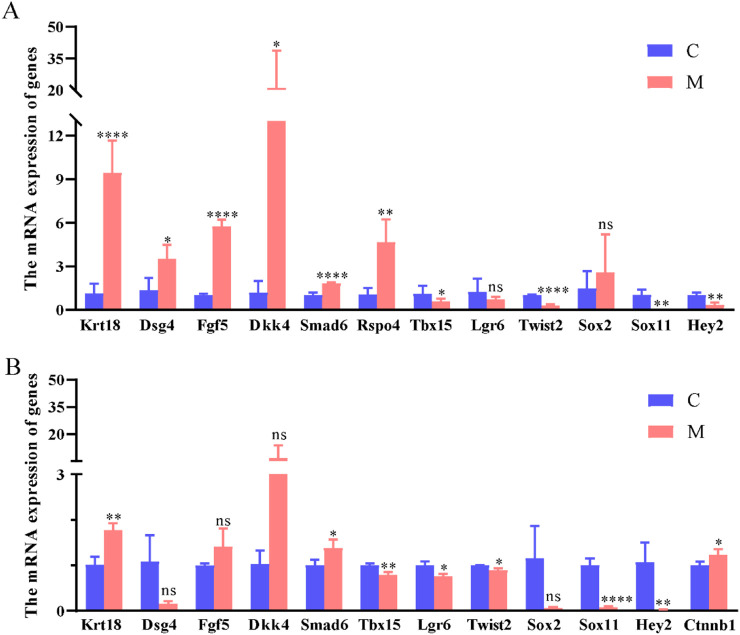
Verification of the DEGs between the medium-concentration anwuligan treatment group and the control group. **(A)** qRT-PCR results of the randomly selected genes between M and C in mouse skin treated with 0 mg/kg and 2 mg/kg anwuligan. **(B)** qRT-PCR results of the randomly selected genes between M and C in HFSCs treated with 0 μmol/L and 10 μmol/L anwuligan.

To verify the results of RNA-seq, 13 differentially expressed genes, including 6 downregulated and 7 upregulated genes, were randomly selected for qRT-PCR assay. The results of qRT-PCR analysis of these genes in mouse dorsal skin tissues are shown in Figure 5. Of these, *Krt18*, *Dsg4*, *Fgf5*, *Dkk4*, *Smad6*, and *Rspo4* were significantly upregulated, while *Tbx15*, *Twist2*, *Sox11*, and *Hey2* were significantly downregulated; in addition, no significant differences were observed between *Lgr6* and *Sox2*(Figure 5A).

To further investigate the expression of *Ctnnb1* and RNA-seq-identified differentially expressed genes in HFSCs, this study treated HFSCs with 0 μmol/L, 10 μmol/L, and 20 μmol/L of anwuligan, followed by RNA extraction and qRT-PCR analysis. The expression of the *Ctnnb1* gene showed no significant difference between 0 μmol/L and 20 μmol/L anwuligan treatment groups (not shown). Based on this, HFSCs treated with 0 μmol/L and 10 μmol/L anwuligan were used for further qRT-PCR detection. The qRT-PCR results revealed that *Krt18*, *Smad6*, and *Ctnnb1* were significantly upregulated, while *Tbx15*, *Lgr6*, *Twist2*, *Sox11*, and *Hey2* were significantly downregulated; in addition, no significant differences among *Dsg4*, *Fgf5*, *Dkk4*, and *Sox2* were observed (Figure 5B).

**FIGURE 6:**
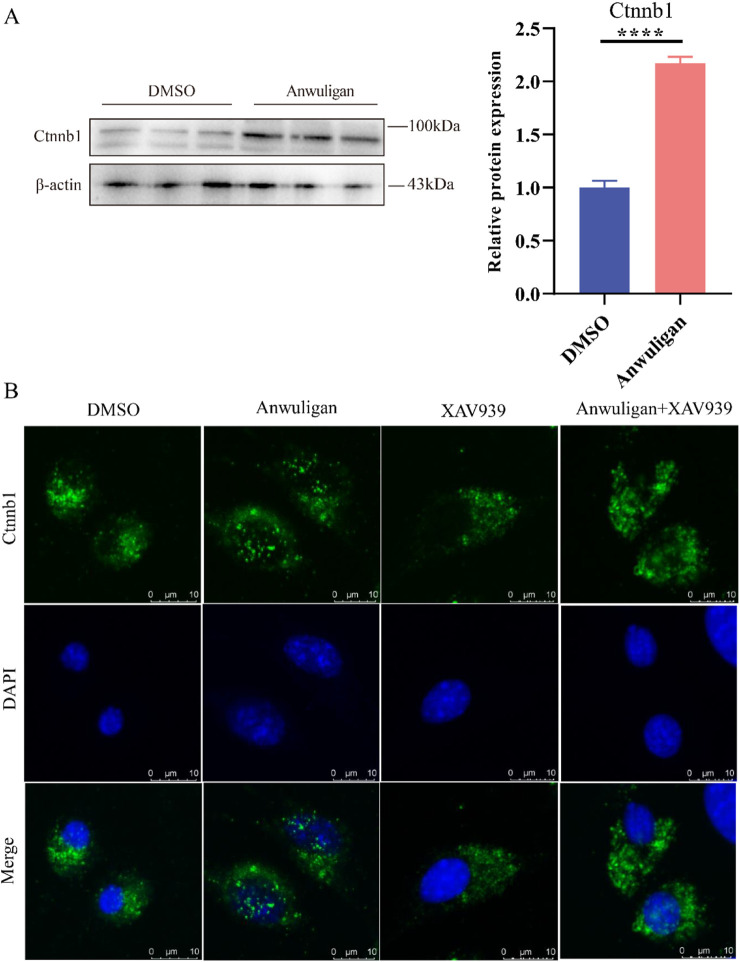
Anwuligan promotes Ctnnb1 expression and nuclear localization. **(A)** Western blot detection of protein expression levels for Ctnnb1. **(B)** Immunofluorescence detection of nuclear localization of Ctnnb1.

As mentioned above, we found that *Ctnnb1* was not significantly different by RNA-seq analysis but significantly upregulated in HFSCs treated with anwuligan (Figure 5). Western blot (Figure 6A) analysis also confirmed that the expression of Ctnnb1 was significantly upregulated in anwuligan-treated HFSCs. Meanwhile, immunofluorescence (Figure 6B) results revealed that anwuligan treatment promoted the nuclear localization of Ctnnb1 in HFSCs, and this effect was inhibited by the Wnt pathway inhibitor XAV939. Genes *Dkk4* and *Smad6* related to the Wnt/β-catenin signaling pathway were differentially expressed in both anwuligan-treated mouse skin tissues and HFSCs. *Lgr6* was differentially expressed in anwuligan-treated HFSCs (Figure 5). The RNA-seq results revealed that the target genes *lef1* and *Tcf7* of Wnt/β-catenin were highly expressed in the anwuligan 2 mg/kg group (Supplementary materials). *Dvl1*, *Dvl2*, and *Dvl3*, the positive regulators of the Wnt/β-catenin signaling pathway, were highly expressed in the anwuligan 2 mg/kg group (Supplementary Materials). The expression of Wnt pathway inhibitors *Sfrp4*, *Sfrp5*, and *Dkk1* was significantly decreased in the anwuligan 2 mg/kg group (Supplementary Materials). These results suggest that anwuligan may promote hair regeneration in mice by activating the Wnt/β-catenin signaling pathway via Ctnnb1.

### Anwuligan promotes HFSC proliferation

**FIGURE 7:**
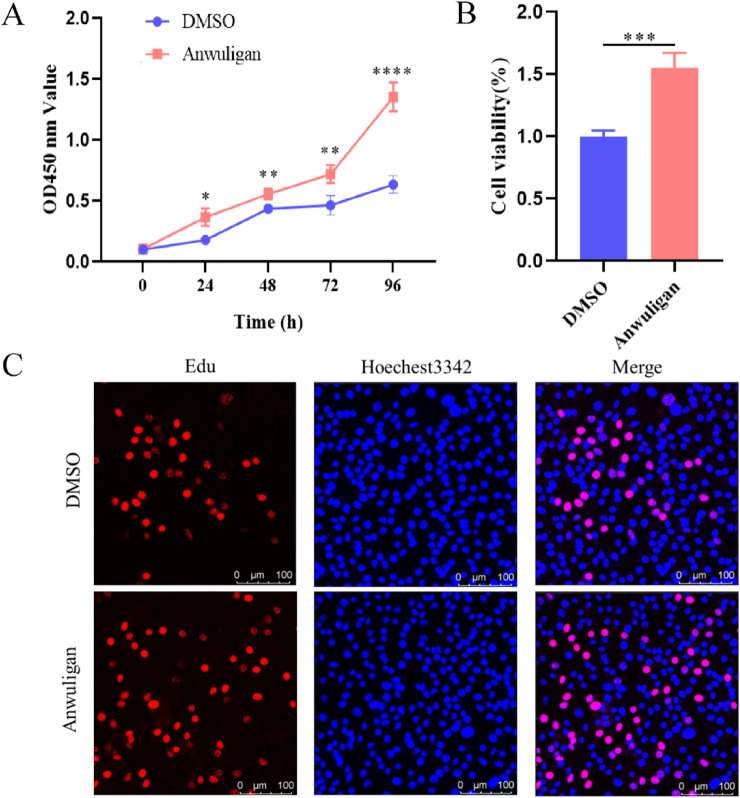
Anwuligan stimulated the proliferation of HFSCs. **(A)** CCK-8 assay. **(B)** MTT assay. **(C)** EdU assay.

In this study, CCK-8, MTT, and Edu assays were utilized to investigate the effect of anwuligan on the biological properties of HFSCs. When reached 70%–80% confluency, HFSCs were treated with 10 μmol/L anwuligan for 48 h and utilized for CCK-8, MTT, and EdU assays. The results of MTT and CCK-8 assays indicated that the cell viability of HFSCs increased significantly after anwuligan treatment (Figures 7A,B). The results of EdU assay revealed that the number of EdU-positive HFSCs increased significantly after anwuligan treatment (Figure 7C). These results indicated that anwuligan could promote the proliferation of HFSCs.

## Discussion

Hair loss has emerged as a prevalent issue affecting both middle-aged and younger populations. It is a multifactorial condition influenced by a complex interplay of genetics, nutrition, disease, medications, physical and chemical damage, and stress (Arck et al., 2003; Heilmann-Heimbach et al., 2017; Almohanna et al., 2019). In TCM theory, hair loss is frequently associated with specific pathological patterns, including “kidney deficiency,” “blood deficiency,” “dampness-heat,” and “liver qi stagnation.” TCM approaches aim to improve the hair follicle microenvironment and promote hair regrowth by nourishing the liver and kidney, promoting blood flow, eliminating blood stasis, and addressing imbalances like heat and dampness. Moreover, TCM not only exhibits pharmacological activity that stimulates hair growth but also regulates hair follicle abnormalities through multi-pathway, multi-target, and multi-channel mechanisms. Currently, the treatment of hair loss is shifting from symptom control to mechanism-based interventions, with recent breakthroughs in Wnt pathway modulation and immunotherapy showing promising potential for more effective solutions.

Anwuligan, a monomeric lignan from forsythia, was recently shown to inhibit non-small-cell lung cancer (Niu et al., 2023). However, its relationship with hair loss remains virtually unknown. In this study, we treated mice with anwuligan and then collected their skin tissues for RNA-seq. RNA-seq analysis revealed that DEGs between 0 mg/kg and 2 mg/kg anwuligan-treated mice were significantly enriched in several key pathways, including metabolic pathways, protein digestion and absorption, retinol metabolism, MAPK signaling pathway, PI3K–Akt signaling pathway, Wnt signaling pathway, PPAR signaling pathway, BMP signaling pathway, and TGF-beta signaling pathway. We found that these pathways were involved in hair follicle development (Wang et al., 2022). As discussed, the DEGs were significantly enriched in metabolic pathways and protein digestion and absorption. This is because hair follicle regeneration is a dynamic process critically reliant on energy metabolism and cellular metabolic reprogramming, wherein the fate of HFSCs is determined by the coordinated regulation of glucose, lipid, and amino acid metabolism (Kim et al., 2020). Wnt and Bmp signaling pathways play essential roles in regulating the quiescence and activation of HFSCs (Plikus et al., 2008; Yi, 2017). Vitamin A (primarily as retinol) coordinates HFSC fate by modulating its levels, directing stem cells toward either wound healing or hair growth (Tierney et al., 2024). Furthermore, the PPAR signaling pathway plays a critical role in hair follicle development (Icre et al., 2006; Bai et al., 2022).

Among the DEGs identified in this study, *Dkk4* and *Smad6* were altered in both anwuligan-treated mouse skin tissues and HFSCs, while *Lgr6* was particularly differentially expressed in anwuligan-treated HFSCs. *Dkk4*, a key antagonist of the Wnt/β-catenin signaling pathway, plays a precise regulatory role in hair follicle development and regeneration (Khatif and Bazzi, 2021; 2023). *Lgr6*, a Wnt pathway co-receptor predominantly expressed in HFSCs, enhances Wnt/β-catenin signaling to support hair follicle development and regeneration (Snippert et al., 2010; de Lau et al., 2011; Chen et al., 2023). *Smad6*, an inhibitor of the BMP signaling pathway, plays a critical role in epithelial stem cell maintenance and progenitor cell differentiation (Rendl et al., 2008; Li et al., 2024). The core components of the Wnt/β-catenin signaling pathway include receptors FZD and LRP5/6, cytoplasmic transducers DvL, AXIN, APC, GSK-3β, and CK-1α, the key effector β-catenin, and the transcription factor TCF/LEF (Foord et al., 2005; Liu et al., 2022). Disheveled paralogs positively regulate the Wnt/β-catenin signaling pathway (Wang et al., 2015). Our RNA-seq results demonstrated that *Dvl1*, *Dvl2*, and *Dvl3* were highly expressed. The Wnt/β-catenin signaling pathway target genes *Lgr6*, *lef1*, and *Tcf7* (Lien et al., 2014) showed elevated expression, while the expression of Wnt inhibitors *Sfrp4*, *Sfrp5*, and *Dkk1* (Huyghe et al., 2015; Guo et al., 2019) was significantly reduced in anwuligan-treated mouse skin. These findings corroborate our RNA-seq results.

Our network pharmacology analysis suggested that anwuligan promotes hair follicle regeneration by activating the Wnt/β-catenin signaling pathway, potentially through direct binding to CTNNB1. This prediction was experimentally confirmed through Western blot analysis and immunofluorescence assay. Previous genetic studies have shown that conditional knockout of *Ctnnb1* in the skin impedes the transition of hair follicles from the resting phase to the growth phase, leading to arrested hair growth (Deschene et al., 2014). In this study, we observed that *Ctnnb1* is significantly upregulated in anwuligan-treated HFSCs, but this effect was not observed in skin tissues. The cellular complexity of skin tissue and the sparse distribution of HFSCs likely explain this result. Importantly, Ctnnb1 expression is spatiotemporally restricted, being detectable only in specific niches, such as in the hair germ during early anagen (Lien et al., 2014), which would be diluted in a whole-tissue lysate.

HFSCs are the “engine” and “command center” of hair follicle regeneration. HFSCs are located in the bulge region and the inner and outer root sheaths at the junction of the arrector pili muscle and the hair follicle, and they are involved in the periodic regeneration of hair follicles (Zhang and Chen, 2024). The Wnt/β-catenin signaling pathway is closely related to the cyclical regeneration of hair follicles, wound healing, scar formation, and proliferation (Choi et al., 2013) and is a molecular signaling pathway closely related to HFSCs (Du et al., 2018). When HFSCs receive Wnt signals, the β-catenin signaling pathway is activated, causing HFSCs to switch from a quiescent state to a proliferative state, initiating the regeneration program (Hsu et al., 2011; Lien and Fuchs, 2014). The activated HFSCs begin to proliferate extensively and migrate downward, providing a large source of cells for the construction of new hair follicle structures (Hsu et al., 2011).

Anwuligan is one of the active chemical components in the TCM herb *Forsythia suspensa*. Forsythia has been extensively cultivated in the Changzhi region of Shanxi Province, with its products holding over 30% of the national market share, which has a significant industrial standing. Recent studies have revealed that multiple Chinese herbal medicines possess potential for promoting hair regeneration, such as *Polygonum multiflorum*, *Platycladus orientalis* (leaf), *Scutellaria baicalensis*, and ginger. Several herbs show potential for hair growth but face significant challenges. The *Polygonum multiflorum* extract promotes hair growth, but its anthraquinones cause hepatotoxicity, limiting its safe use (Shin et al., 2020; Gong et al., 2024). Although the *Platycladus orientalis* leaf extract and baicalin stimulate hair growth, the former faces formulation stability issues (Kim et al., 2024; Hong et al., 2025). Conversely, 6-gingerol inhibits hair growth (Miao et al., 2013). Critically, these findings remain largely confined to cellular and *in vitro* models. This study demonstrates the hair-regenerative effects of anwuligan using an animal model, which more closely mimics the *in vivo* environment and provides more compelling evidence than *in vitro* studies. The use of a single compound also provides unequivocal evidence of its efficacy, free from the confounding effects of other substances.

In our study, we identify a previously unrecognized role of anwuligan in promoting hair regeneration through the Wnt/β-catenin pathway. However, the present study still has some shortcomings. For example, the ETCM 2.0 database used in our research is human-specific and incomplete, which could introduce certain limitations and biases. Furthermore, the limited sample size in our animal study must be considered a potential constraint on the statistical validity and generalizability of the RNA-seq results. Additionally, the biological differences in genetic background and hair follicle development between mice and humans may limit the translational relevance of anwuligan for treating hair loss. Therefore, a clinical trial should be undertaken to verify the effects of anwuligan in humans. Our findings establish a foundation for innovative hair loss treatment strategies, and anwuligan may emerge as a candidate single-molecule drug for the treatment of hair loss.

In contrast, anwuligan exhibits multifaceted biological activities, including antioxidant, anti-inflammatory, antiviral, and anticancer effects. As a plant-derived natural product, it possesses excellent biocompatibility and low toxicity and may directly target the Wnt/β-catenin signaling pathway, which is critical for hair follicle development and regeneration. In summary, anwuligan could potentially offer broader applications and translational prospects in the field of hair regeneration.

## Conclusion

**FIGURE 8:**
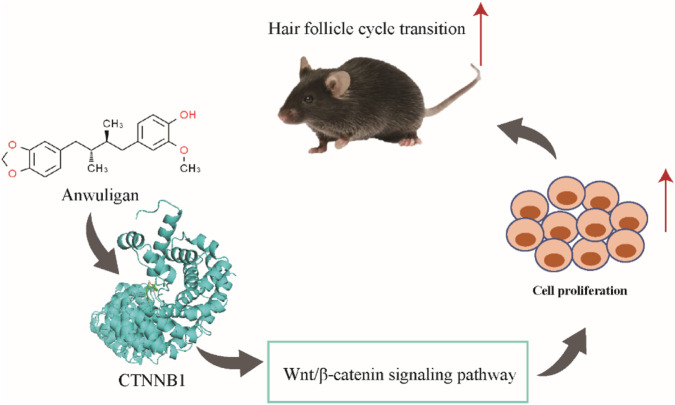
The proposed model of anwuligan promotes the transition of the hair follicle cycle via the Wnt/β-catenin signaling pathway.

In this study, we demonstrated that anwuligan promotes hair regeneration in C57BL/6 mice. The expression of Wnt/β-catenin signaling pathway-related genes in C57BL/6 mouse skin tissues and HFSCs was significantly affected by anwuligan treatment, while anwuligan also significantly enhanced the proliferation of HFSCs. The above results indicate that anwuligan may promote the transition of the hair follicle cycle via the Wnt/β-catenin signaling pathway (Figure 8).

## Data Availability

TThe data presented in the study are deposited in the NCBI-SRA (https://www.ncbi.nlm.nih.gov/sra/) repository, accession number PRJNA1278808.
